# SOX3 promotes generation of committed spermatogonia in postnatal mouse testes

**DOI:** 10.1038/s41598-020-63290-3

**Published:** 2020-04-21

**Authors:** Dale McAninch, Juho-Antti Mäkelä, Hue M. La, James N. Hughes, Robin Lovell-Badge, Robin M. Hobbs, Paul Q. Thomas

**Affiliations:** 1 School of Biological Sciences and Robinson Research Institute, University of Adelaide, Adelaide, SA 5005 Australia; 2 Australian Regenerative Medicine Institute, Monash University, Melbourne, VIC 3800 Australia; 3 Development and Stem Cells Program, Monash Biomedicine Discovery Institute and Department of Anatomy and Developmental Biology, Monash University, Melbourne, VIC 3800 Australia; 4 Present Address: Institute of Biomedicine, University of Turku, Turku, Finland; 5 Laboratory of Stem Cell Biology and Developmental Genetics, The Francis Crick Institute, London, NW1 1AT UK

**Keywords:** Spermatogenesis, Stem-cell differentiation

## Abstract

SOX3 is a transcription factor expressed within the developing and adult nervous system where it mostly functions to help maintain neural precursors. *Sox3* is also expressed in other locations, notably within the spermatogonial stem/progenitor cell population in postnatal testis. Independent studies have shown that *Sox3* null mice exhibit a spermatogenic block as young adults, the mechanism of which remains poorly understood. Using a panel of spermatogonial cell marker genes, we demonstrate that *Sox3* is expressed within the committed progenitor fraction of the undifferentiated spermatogonial pool. Additionally, we use a *Sox3* null mouse model to define a potential role for this factor in progenitor cell function. We demonstrate that *Sox3* expression is required for transition of undifferentiated cells from a GFRα1+ self-renewing state to the NGN3 + transit-amplifying compartment. Critically, using chromatin immunoprecipitation, we demonstrate that SOX3 binds to a highly conserved region in the *Ngn3* promoter region *in vivo*, indicating that *Ngn3* is a direct target of SOX3. Together these studies indicate that SOX3 functions as a pro-commitment factor in spermatogonial stem/progenitor cells.

## Introduction

SOX3 is a member of the SOX (Sry-related HMG box) family of transcription factors (TFs), of which there are 20 members in mammals. SOX TFs are expressed within many tissues of the mouse embryo and regulate a range of important cellular activities including self-renewal, specification and differentiation^1,2^. SOX TFs bind to variants of the SOX consensus motif (A/TA/TCAAA/TG) *via* a highly conserved HMG domain that shares at least 50% sequence identity with the founding member SRY. *Sox*1*, Sox2* and *Sox3* have high similarity across their entire open reading frame and together comprise the *SoxB1* subgroup. *SoxB1* genes are expressed in neural progenitor cells throughout the entire vertebrate neuroaxis and are generally down-regulated during differentiation^3,4^. Loss-of-function and overexpression experiments in a range of vertebrate systems indicate important and overlapping roles for SOXB1 factors in the generation and maintenance of neural stem/progenitor cells^5–8^. SOX3 is also expressed in progenitor cells outside of the nervous system, including the postnatal testis. However, the role of SOX3 in stem/progenitor cell maintenance in these tissues is less well understood.

Spermatogenesis is the fundamental biological process required for the generation of sperm from progenitor cells via mitosis, meiosis, and a complex program of cellular differentiation. Importantly, in mammals, as in many other animals, sustained spermatogenesis in the adult is dependent on a resident population of germline cells with self-renewal potential. In the mouse testis, this stem cell activity is contained within a heterogeneous population of germ cells known as undifferentiated spermatogonia that develop from gonocytes (foetal germ cells) during the first week of postnatal development. The undifferentiated pool is located in the basal layer of the seminiferous tubules, and is composed of cells of distinct topologies; isolated type A-single spermatogonia (A_s_) and interconnected chains of 2 or more cells formed from incomplete cytokinesis during cell division referred to as A-paired (A_pr_) and A-aligned (A_al_) spermatogonia, respectively^9^. Upon commitment to differentiate, cells convert to type A1 spermatogonia, which then undergo a series of rapid mitotic divisions prior to meiosis and sperm formation. Besides having distinct cell division kinetics, differentiating spermatogonia can be distinguished from undifferentiated cells by expression of the receptor tyrosine kinase c-KIT plus DNA methyltransferases 3A and 3B (DNMT3A/DNMT3B)^10,11^.

All cells within the undifferentiated pool may possess self-renewal potential^12^. However, only a small subset of this population act as stem cells in the steady-state tissue, with a majority of undifferentiated cells being primed to differentiate and therefore acting as committed progenitor/transit-amplifying cells^13^. The fate tendencies of undifferentiated cells correlate with gene expression patterns and chain length. Specifically, steady-state stem cells express *Gfra1*, encoding a co-receptor for the key niche-derived growth factor glial cell line derived neurotrophic factor (GDNF) and exist primarily as A_s_, A_pr_ and some short-chained A_al_ cells^14^. A primitive subset of GFRα1+ A_s_ and A_pr_ spermatogonia with potent stem cell activity and marked by expression of transcription factors EOMES and PDX1 has also recently been described^15,16^. In contrast, the majority of A_al_ spermatogonia express *Ngn3* and *Rarg* and are usually differentiation-committed^17–20^. Interestingly, lineage-tracing studies have demonstrated that a small fraction of the NGN3 + population is still capable of forming stable long-lived clones within the testis^19^. Moreover, NGN3 + A_al_ cells occasionally fragment to shorter chains plus A_s_ cells and may revert gene expression patterns to a GFRα1+ state, demonstrating the dynamic nature of the stem cell pool^16,21^. This limited contribution of NGN3 + cells to the steady-state self-renewing pool is also enhanced under conditions of tissue regeneration^19^. However, in contrast to GFRα1+ spermatogonia, NGN3/RARγ + undifferentiated cells are sensitive to retinoic acid, a key endogenous differentiation stimulus, which promotes a differentiation-committed fate^18^.

As transition from the GFRα1+ to NGN3 + state switches the predominant fate of undifferentiated cells from self-renewal to differentiation, it must be tightly regulated to ensure tissue homeostasis. A limited number of factors have been directly implicated in regulation of this transition. For instance, the SOHLH1/2 transcription factors and mTORC1-signalling pathway promote exit from a GFRα1+ state while the NANOS2 RNA binding protein prevents the GFRα1+ to NGN3 + transition *via* direct inhibition of both *Sohlh2* mRNA translation and mTORC1 activation^20,22–25^. Despite the importance of such factors and pathways in fate transitions within the undifferentiated pool, the relevant downstream effectors remain poorly characterised.

*Sox3* is one of a number of identified target genes of SOHLH1/2 within the testis and is reported to play a role in spermatogenesis, whereby *Sox3* deletion causes a block in spermatogenesis that is most severe in mice bred on the C57Bl/6 genetic background^23,26,27^. However, the exact nature of this spermatogenic block and the underlying molecular mechanisms are not fully understood. Through use of a *Sox3*-GFP knock-in mouse model we now confirm that *Sox3* is specifically expressed within the committed/differentiation-destined progenitor fraction of the undifferentiated pool and we define its critical role in the GFRα1+ to NGN3 + spermatogonial transition.

## Results

### SOX3 expression is restricted to committed spermatogonial progenitor cells

**Figure 1:**
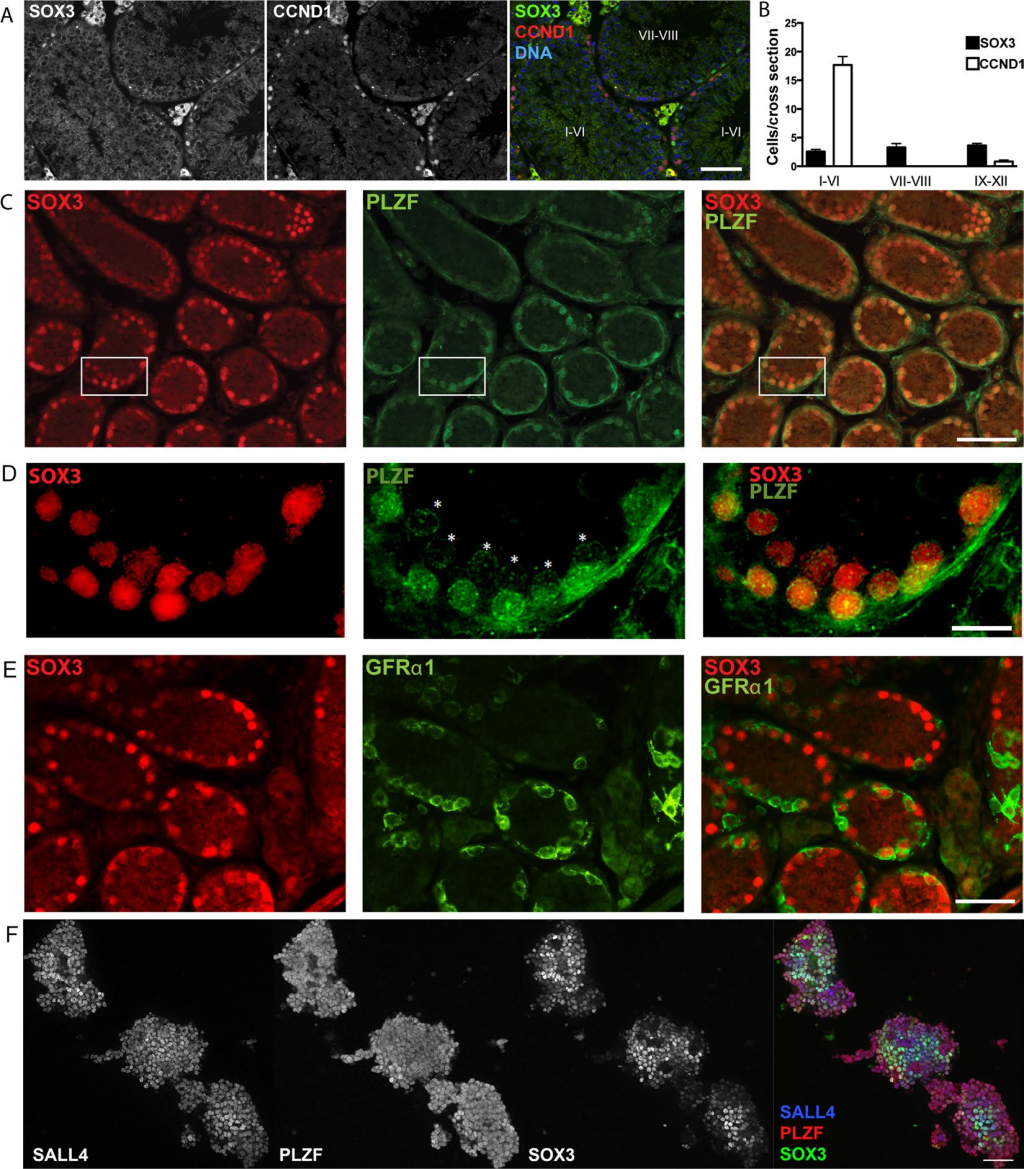
SOX3 expression is found within a subset of undifferentiated spermatogonia. (**A**) IF of adult testis cross sections showing expression of SOX3 is restricted to spermatogonia. Note the staining in Leydig cells is non-specific. (**B**) The number of SOX3 + spermatogonia remains constant, independent of the stage of seminiferous epithelium cycle, whereas CCND1 + spermatogonia are more prevalent in stage I-VI tubules. (**C**) IF of SOX3 and PLZF in P7 testis cross sections demonstrates an extensive overlap, with only a few SOX3-/PLZF + spermatogonia present. (**D**) 60X magnification of white box in (**C**) highlighting variations in PLZF expression from low (*) to high. (**E**) IF of SOX3 and GFRα1 in P7 testis cross-sections showing limited overlap of expression. (**F**) IF of SOX3, PLZF and SALL4 in cultures of undifferentiated spermatogonia derived from juvenile mice, PLZF and SALL4 are detected within essentially all cells, while SOX3 is only present within a subset of cells. Scale bars are 50μm. (**A**,**C**,**E**&**F**), 10μm (**D**).

Previous studies have shown that *Sox3* expression in the testis is restricted to spermatogonial populations within the basal layer of the seminiferous tubules^8,26,27^. Through use of a SOX3-specific antibody^4^, we confirmed by immunofluorescence (IF) analysis that SOX3 protein is restricted to spermatogonia of wild type (WT) adult testis (Fig. 1A). Spermatogenesis occurs in a coordinated, cyclic process that can be divided into 12 stages in the mouse. Sections of tubules at a given stage contain defined populations of spermatogonia, spermatocytes and developing spermatids at distinct differentiation and maturation steps^9,28^. Importantly, SOX3-positive spermatogonia were present at all stages of the seminiferous epithelium cycle, indicating that *Sox3* is expressed in the undifferentiated population (Fig. 1B). Moreover, spermatogonia expressing *Sox3* displayed low levels of Cyclin D1 (*Ccnd1*), a marker predominantly expressed by differentiating spermatogonia (Fig. 1A)^29,30^. In contrast to the stage-independent presence of SOX3-positive cells, spermatogonia expressing CCND1 were predominantly found in stage I-VI tubules in which populations of differentiating spermatogonia are the most abundant (Fig. 1B).

In order to better define the identity of the SOX3-positive population we next performed IF analysis of prepubertal, WT postnatal day 7 (P7) testis, which is relatively enriched in undifferentiated spermatogonia. Comparison of SOX3 expression with that of promyelocytic leukaemia zinc finger (PLZF), which is expressed throughout the undifferentiated pool^31,32^, revealed extensive overlap (Fig. 1C,D). Analysis of adult and juvenile testis therefore suggested that SOX3 is broadly expressed in the undifferentiated population. To investigate this further, we compared SOX3 expression with that of GFRα1, a marker for the self-renewing fraction of undifferentiated spermatogonia^14^. In comparison to the pronounced co-expression of SOX3 with PLZF, we found a limited overlap in expression of SOX3 and GFRα1 (Fig. 1E), suggesting that within the undifferentiated population, SOX3 is preferentially expressed in the committed progenitor fraction^23^.

To further characterise the expression of SOX3 in spermatogonia, we also performed IF analysis on cultures of undifferentiated spermatogonia derived from juvenile WT mice^31^. SOX3 expression was compared to that of PLZF and the transcription factor SALL4, which is expressed in undifferentiated and differentiating spermatogonia^33^. Consistent with previous studies, PLZF and SALL4 were expressed by essentially all cells within the colonies of cultured cells (Fig. 1F)^33^. In contrast, SOX3 expression was clearly heterogeneous. Given that these cultures are considered to contain a mix of both stem and committed progenitor cells^34^, our data support the conclusion that SOX3 expression is restricted to a subset of undifferentiated cells, likely representing the committed progenitor fraction, consistent with previous reports ^8,26,27^.

**Figure 2:**
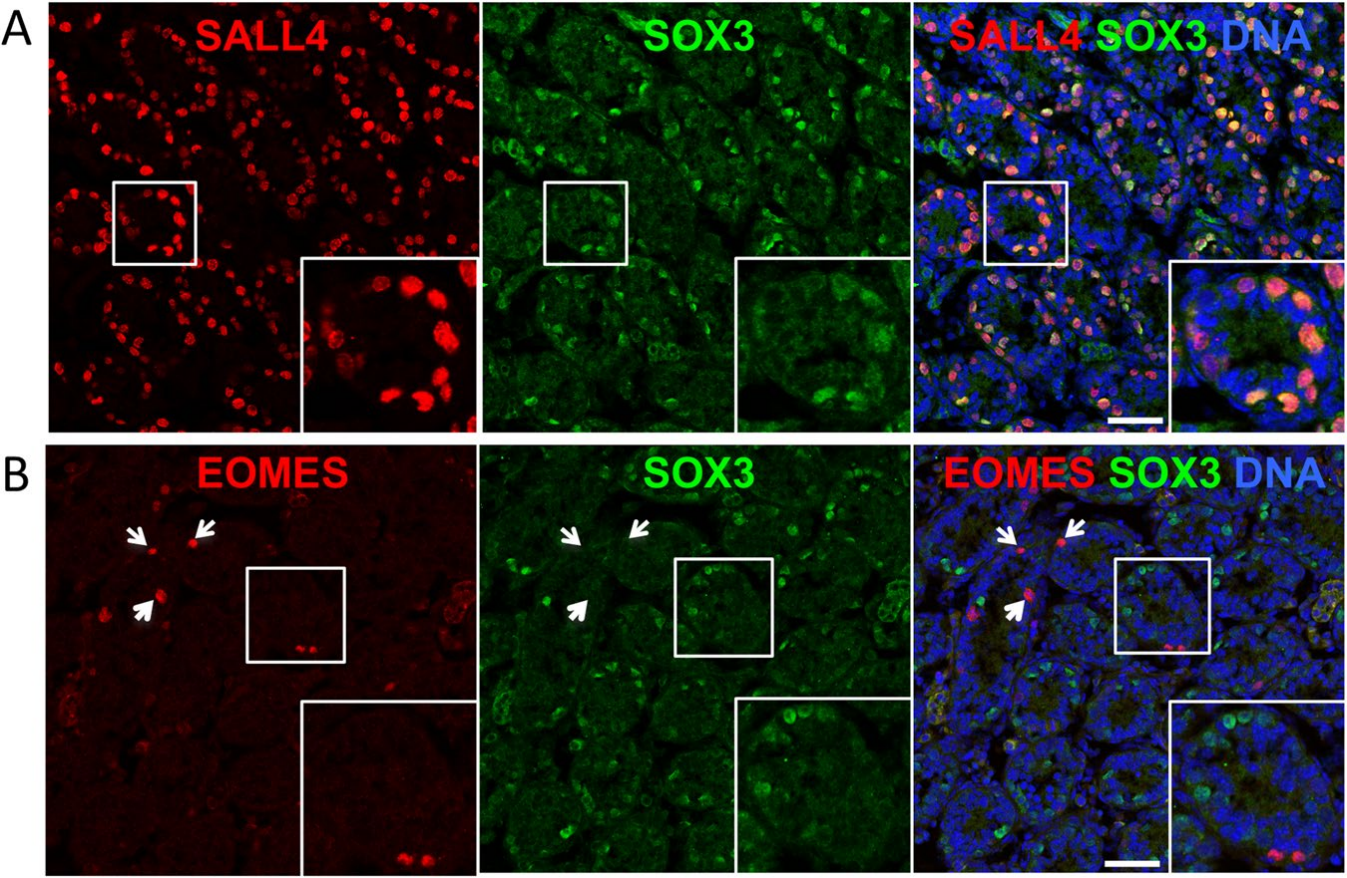
Characterising the expression of SOX3 in juvenile testis. (**A**,**B**) Representative IF analysis of SOX3 and markers of spermatogonia (SALL4) and primitive self-renewing cells (EOMES) in testis sections from P7 mice (n = 3 mice). Insets show higher magnification details of indicated regions. Arrowheads indicate EOMES + SOX3- spermatogonia. Scale bars are 50μm.

Notably, from IF analysis of P7 testis sections, we confirmed that essentially all SOX3 + cells were positive for spermatogonial marker SALL4 but that (SOX3) expression was restricted to a subset of the SALL4 + population (32.7 ± 8.0% of SALL4 + cells were SOX3 + , mean ± s.e.m, *n* = 3 mice,>70 tubule cross-sections scored per sample) (Fig. 2A). In contrast, no overlap in expression was found between SOX3 and EOMES, a marker of the most primitive cells within the GFRα1+ self-renewing fraction (Fig. 2B)^15,16^. Combined, our results indicate that (SOX3) expression delineates a subset of differentiation committed/primed spermatogonia.

**Figure 3:**
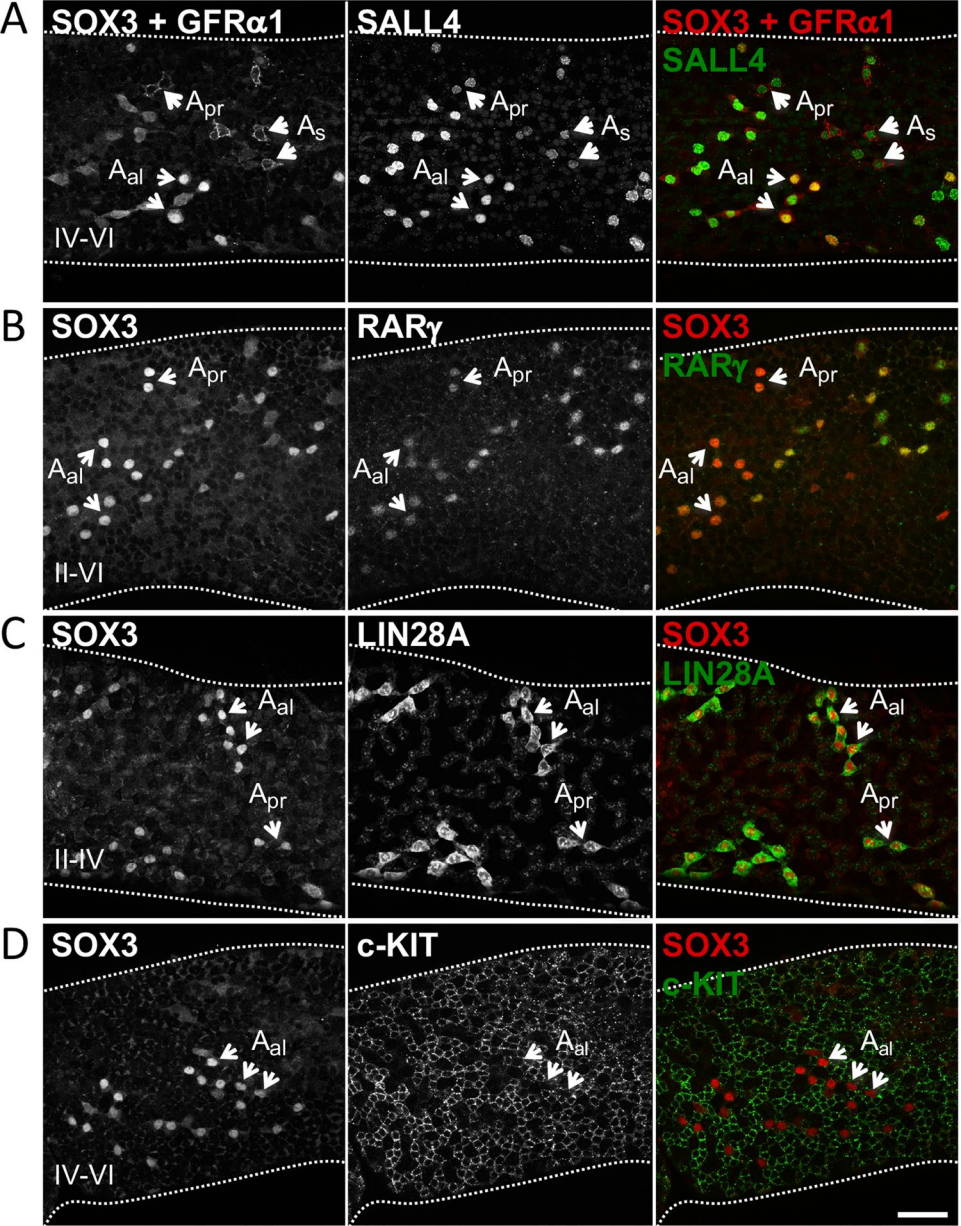
Characterising the expression of SOX3 in adult testis. Assessing co-expression of SOX3 with a panel of undifferentiated and differentiated spermatogonia cell markers in adult seminiferous tubules by wholemount IF. SOX3 shows limited overlap in expression with stem cell-associated marker GFRα1 (**A**) but is co-expressed with progenitor markers RARγ (**B**) and LIN28A (**C**) within the undifferentiated pool. SOX3 is expressed at low levels or is undetectable in mid-late stage differentiating spermatogonia marked with c-KIT (**D**). Note that antibodies to SOX3 and GFRα1 are both raised in goat and detected in the same fluorescence channel. Cell staining patterns are used to distinguish GFRα1 (cell membrane) and SOX3 (nucleus). Selected undifferentiated cells and stages of tubules are indicated. Representative images are shown (n = 4 mice). Scale bars are 50μm.

Finally, to confirm the expression pattern of SOX3 within mature stem and progenitor spermatogonial populations, we compared its expression with that of a number of distinct spermatogonial markers by wholemount analysis of adult seminiferous tubules (Fig. 3). Importantly, IF for GFRα1 and SOX3 in adult tubules demonstrated that expression of these proteins largely marked distinct spermatogonial populations, in agreement with our previous results (Fig. 1E). GFRα1 primarily labelled A_s_ and A_pr_ cells while SOX3 was present in GFRα1-negative SALL4-positive A_al_ (Fig. 3A). Antibodies to GFRα1 and SOX3 used in wholemount analysis are both raised in goat and are visualized in the same fluorescence channel but can be distinguished by cell membrane vs. nuclear staining pattern (Fig. 3A and S1). Rather, we found that SOX3 was co-expressed in chains of undifferentiated spermatogonia plus cells at early differentiation stages with genes that mark committed progenitor fractions, including RARγ and LIN28A (Fig. 3B,C and data not shown)^18,32^. In contrast, chains of c-KIT + spermatogonia at mid-to-late stages of differentiation (A_3_ to B) expressed low or undetectable levels of SOX3 (Fig. 3D)^10,11,16,30^.

Taken together, our data demonstrate that SOX3 expression is selectively upregulated upon transition from the self-renewing to the differentiation-primed/committed progenitor state within the undifferentiated population of both juvenile and adult testes. It is subsequently downregulated as they differentiate further.

### Early postnatal defect in 129/Svj *Sox3* null testes

Spermatogenesis phenotypes have been reported in *Sox3* null mice on mixed and C57BL/6 inbred backgrounds^8,26,35^. To investigate whether spermatogenesis is also affected on a 129/Svj inbred background, *Sox3* null mice were backcrossed and examined for testis abnormalities. No significant difference in testis weight or morphology (Fig. S2A,B) was detected in *Sox3* null mice at postnatal day 7 (P7; Fig. S2A,B; WT = 5.3 ± 0.2 mg; KO = 5.1 ± 0.4 mg). Histological analysis also failed to reveal any gross defects at this stage (Fig. S3). In contrast, *Sox3* null testes were significantly smaller than WT testis at P14, P21 (Fig. S2C–F) and at 6 months of age. Histological analysis revealed an abundance of germ cell-depleted tubules in *Sox3* null testes consistent with previous reports^8,26,35^ (Fig. S3E,F). To address whether elevated programmed cell death might account for testis hypoplasia in *Sox3* null mice, we performed TUNEL staining on testis sections. No difference in the number of apoptotic cells was observed between WT or *Sox3* null testis at P7 (Fig. S3C,D).

**Figure 4:**
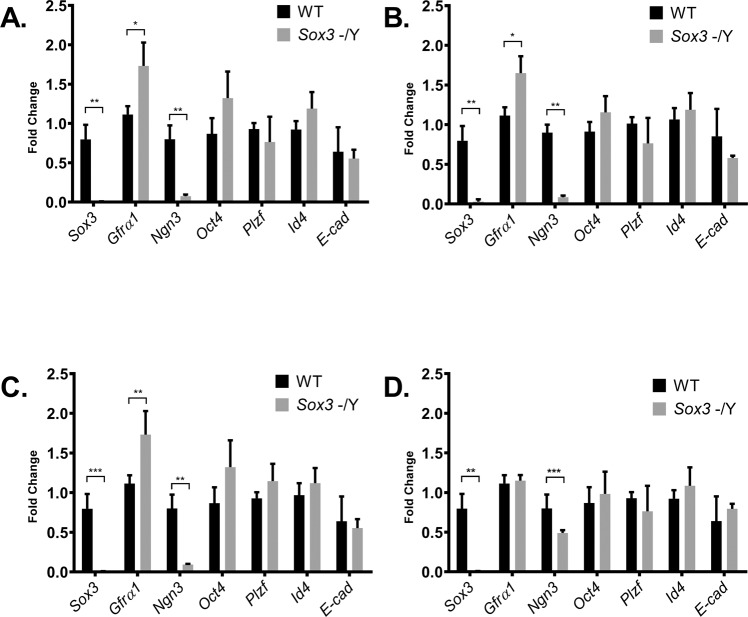
Comparative analysis of *Sox3*^-/Y^ and WT testis gene expression. Expression analysis of spermatogonia cell markers *Sox3*, *Gfra1*, *Ngn3*, *Oct4*, *Plzf*, *Id4* and *E*-*Cad* in P7 (**A**), P14 (**B**), P21(**C**) and P28 (**D**) testes. n = 4 for each genotype, Ρ < 0.05 (*), Ρ < 0.01 (**), Ρ < 0.001 (***) as determined by t-test. Data represented as mean ± s.d.

To further investigate the cellular mechanism that underpins the spermatogenic block in *Sox3* null testes, we compared expression of spermatogonial cell markers in WT and *Sox3* null testis at P7. We reasoned that although *Sox3* null testes were not hypoplastic at this stage, the primary molecular/cellular defect(s) underpinning the defect would likely be present, given that *Sox3* is expressed at this time point (Fig. 1). Expression of *Oct4*/*Pou5f1*, *Plzf*, *Id4* and *Ecad* were not significantly different, suggesting that the number of Type A spermatogonia is not grossly affected by the absence of *Sox3* (Fig. 4A)^31,36^. Further, expression of these markers was not significantly altered in KO testis at P14, P21 and P28. In contrast, *Ngn3*, a marker of differentiation-primed undifferentiated spermatogonia, was substantially reduced at P7 (Fig. 4A) and remained significantly lower throughout postnatal testis development (P14, P21 and P28) (Fig. 4B–D), as observed previously in B6 KO mice^27^. Conversely, expression of *Gfra1*, a marker for the self-renewing fraction of undifferentiated spermatogonia, was significantly higher in *Sox3* null testis compared to WT (Fig. 4). *Gfra1* expression remained significantly elevated at P14 and P21 although it had apparently normalised by postnatal week 4 (Fig. 4B–D). Interestingly, expression of *Id4*, a marker associated with transplantable stem cell activity that is detected in a subset of the GFRα1-positive population in adults, but is more broadly expressed by spermatogonia during postnatal development, was not significantly altered by loss of *Sox3* (Fig. 4A–D)^37^. While the significance of unaltered *Id4* expression is unclear, the increase in *Gfra1* and reduction in *Ngn3* expression in response to *Sox3* deletion suggests a relative increase in self-renewing spermatogonial subsets and a corresponding decrease in differentiation-primed progenitors.

**Figure 5:**
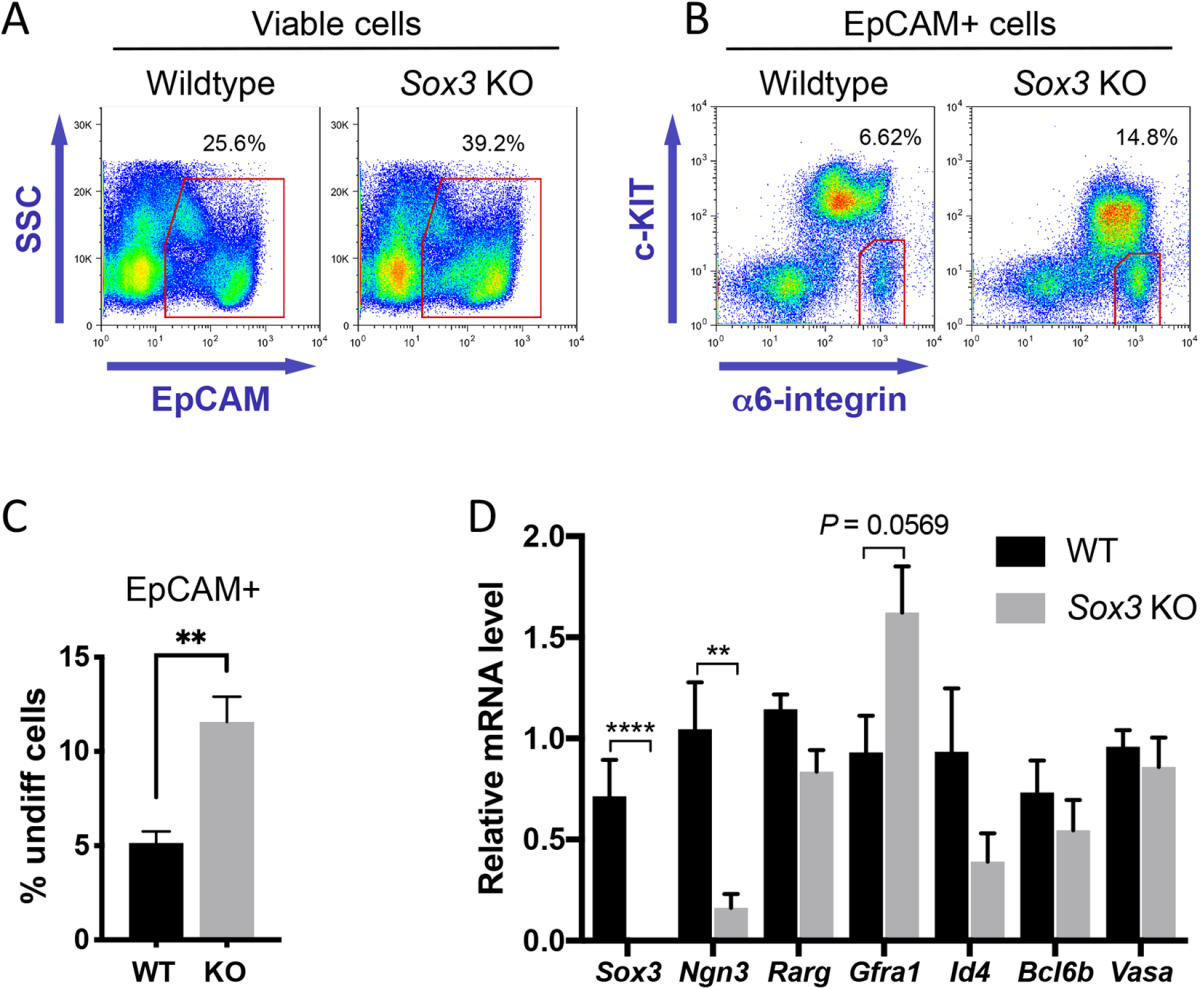
Isolation and analysis of *Sox3* null adult undifferentiated spermatogonia. (**A**,**B**) Flow-sorting strategy for isolation of undifferentiated spermatogonia (EpCAM + c-KIT– α6-integrin+) for gene expression analysis. Percentages of cells within gates are indicated. (**C**) Graph shows percentage of EpCAM+ germ cells within c-KIT– α6-integrin+ undifferentiated cell gate from flow cytometry analysis of wildtype and *Sox3* null adult testis of A and B. Mean values ± s.e.m. are shown (n = 5 wildtype and n = 6 *Sox3* null mice). Significance was determined by two-tailed unpaired *t*-test, P < 0.01 (**). (**D**) RT-qPCR analysis of sorted undifferentiated spermatogonia (EpCAM + c-KIT– α6-integrin+) from wildtype and *Sox3* null adult testis. Expression levels are corrected to β-actin and normalized to a control sample. Mean values ± s.e.m. shown (n = 5 wildtype and n = 6 *Sox3* null mice). Significance was determined by two-tailed unpaired *t*-test and selected P values are indicated, P < 0.01 (**), P < 0.0001 (****).

To confirm these observations in the mature spermatogonial pool, we isolated undifferentiated cells (EpCAM + α6-integrin+ c-KIT–) from wildtype control and *Sox3* KO adults and analysed gene expression by RT-qPCR (Fig. 5)^38^. Within the EpCAM+ germ cell fraction of *Sox3* KO testis a significantly greater proportion of cells were in the α6-integrin+ c-KIT– undifferentiated cell gate compared to controls (Fig. 5B,C), suggesting that spermatogonial differentiation was disrupted in the *Sox3* KO and undifferentiated cells accumulated with age. In agreement with analysis of total testis extracts during postnatal development, *Sox3* KO adult undifferentiated spermatogonia exhibited significantly lower expression of *Ngn3* while other stem and progenitor-associated markers were mostly comparable to controls. Expression of *Gfra1* was increased in *Sox3* KO undifferentiated cells, although not significantly (*P* = 0.0569), consistent with previous results (Figs. 4 and 5D). Combined, our results support the involvement of SOX3 in stem-to-progenitor transition in the male germline and specifically link SOX3 with expression of *Ngn3*, a marker of differentiation-primed undifferentiated spermatogonia^21^.

### SOX3 promotes exit from the GFRα1-positive spermatogonial state

**Figure 6:**
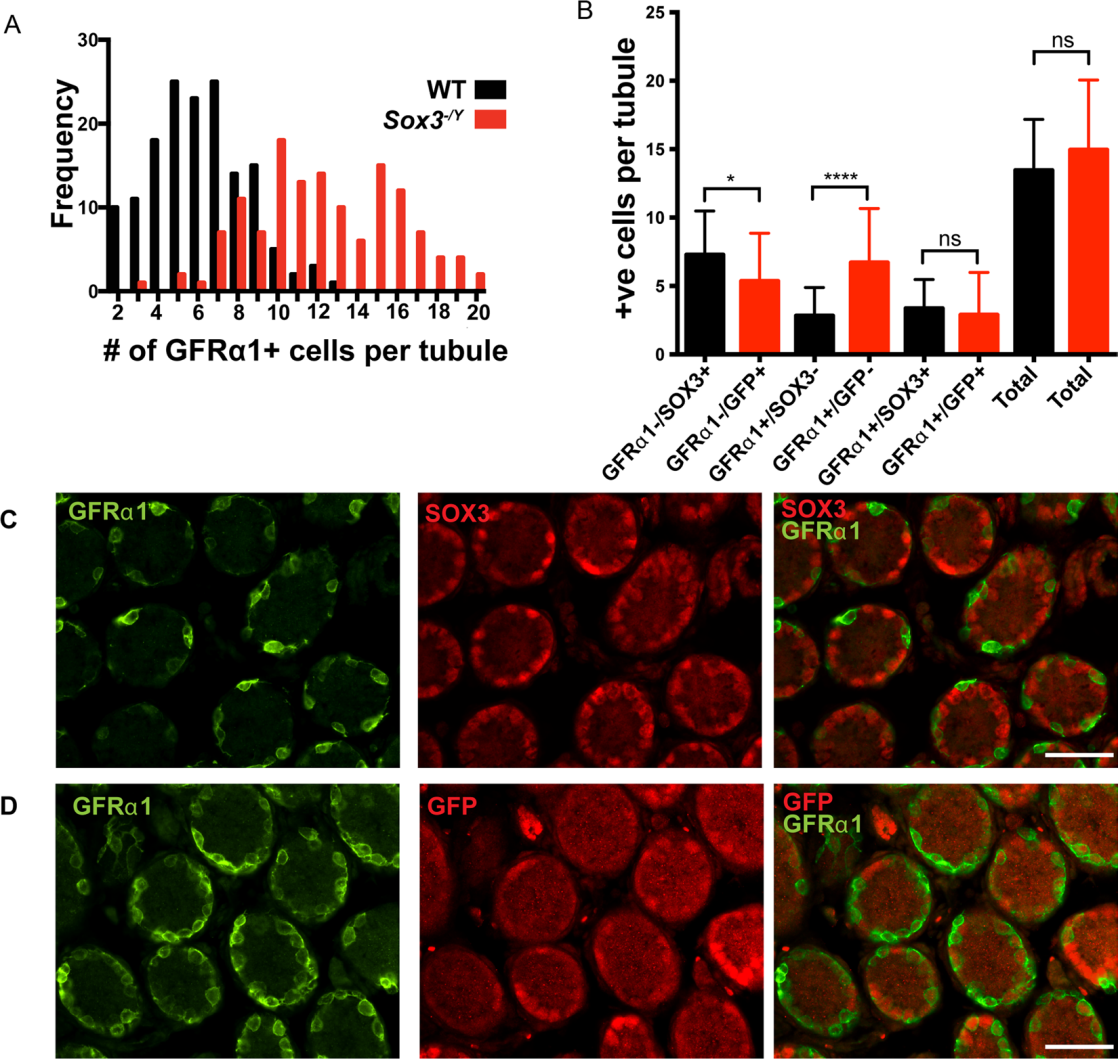
Increased number of GFRα1+ spermatogonia in *Sox3*^−/Y^ testis. (**A**) Frequency plot showing more GFRα1+ cells per tubule in *Sox3* null testis cross-sections (12.3+/− 0.28) compared to WT (6.2+/−0.19), Ρ < 0.0001. (**B**) Cell counts of the different spermatogonia cell identities as marked by SOX3/GFP and or GFRα1 staining. IF of WT testis cross sections at P7 (**C**), and *Sox3* null testis sections (**D**), staining for SOX3 (red) and GFRα1 (green). Minimum 10 fields of view per counted section, 3 sections per testis, n = 3 for each genotype, not significant (ns), Ρ < 0.05 (*), Ρ < 0.0001(****) as determined by Mann-Whitney test. Data represented as mean±s.d., scale bar is 50μm (**C**,**D**).

To further investigate the cellular mechanism that underpins elevated expression of *Gfra1* in *Sox3* null testis, we first performed IF analysis of P7 testis sections. Importantly, we observed a significant increase in the total number of GFRα1 positive cells per tubule cross-section in *Sox3* null testis compared to WT testis (mean of 12.3 ± 0.28 versus 6.2 ± 0.19 cells/tubule, *Ρ* <0.0001; Fig. 6A). This relative expansion of the GFRα1-positive population may suggest that *Sox3* deficiency resulted in a differentiation block at the stem-to-progenitor cell transition or increased self-renewing divisions of-GFRα1-expressing cells. Alternatively, given the dynamics of cell transitions within the undifferentiated compartment^21^, it might represent an inability to maintain a stable progenitor population and increased reversion of committed cells to a self-renewing state.

To investigate these distinct possibilities, we took advantage of a unique feature of our *Sox3*-deficient model. Namely, that *Sox3* deletion is accompanied by insertion of a GFP reporter gene into the *Sox3* locus^8^. Analysis of GFP expression in *Sox3* null testis therefore allows identification of committed progenitor populations that are usually marked by *Sox3* expression in the WT setting. From WT and *Sox3* null testis we therefore scored the number of uncommitted stem cells (GFRα1 + /SOX3- and GFRα1 + /GFP- respectively), transitional/early committed progenitor cells (GFRα1 + /SOX3 + and GFRα1 + /GFP + ) and committed progenitor cells (GFRα1-/SOX3 + and GFRα1−/GFP+) (Fig. 6B). Importantly, no significant difference was detected in the total number of cells expressing these distinct marker combinations. However, significant differences were detected in the relative abundance of two subpopulations. Firstly, the number of cells that exhibited a committed progenitor phenotype (GFRα1-/GFP + *vs*. GFRα1-/SOX3 + ) was significantly reduced in *Sox3* null testes as compared to WT controls. Secondly, there was a significant increase in the number of uncommitted (GFRα1 + /GFP- *vs*. GFRα1 + /SOX3-) stem cells in the *Sox3* null testes. While populations of transitional cells (GFRα1 + /GFP + *vs*. GFRα1 + /SOX3 + ) were not significantly altered upon loss of *Sox3* at this early postnatal age, transitional stem-progenitor cells were found to accumulate in *Sox3* null adults (see below). Our results indicate that while the total population of stem and progenitor spermatogonia is relatively unaffected in the juvenile *Sox3* null testis, there is a shift in balance from stem to committed progenitor cell phenotype. This suggests that *Sox3* promotes the stable transition from stem to progenitor cell states and is consistent with changes in *Gfra1* and *Ngn3* expression in *Sox3* null testis (Figs. 4 and 5).

**Figure 7:**
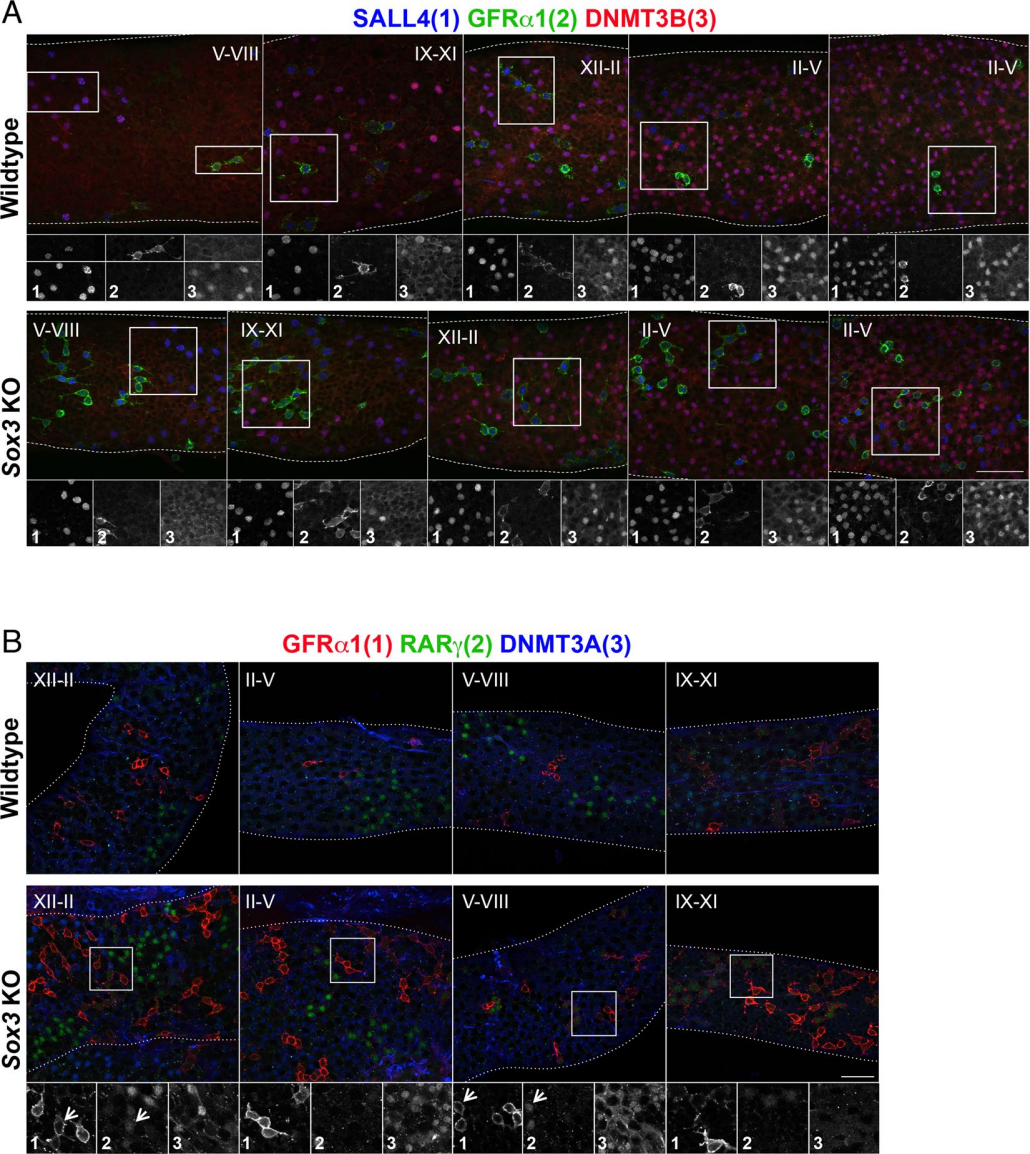
Analysis of *Sox3* null adult seminiferous tubules by whole mount IF. Wholemount IF analysis of WT and *Sox3* knockout adult seminiferous tubules for SALL4, DNMT3B, GFRα1 (**A**) and GFRα1, RARγ, DNMT3A (**B**) at the indicated spermatogenic stages. Grayscale panels show individual immunostaining within the indicated area. Arrowheads in (**B**) indicate GFRα1+/RARγ + transitional cells found relatively frequently in *Sox3* knockout tubules but rarely in WT samples. Scale bars are 50μm.

To further investigate this phenotype and the consequences of inefficient stem to progenitor cell conversion on the spermatogenic pathway, we analysed *Sox3*-deficient adult testis by whole mount IF of seminiferous tubules (Fig. 7A). In this analysis, SALL4 was used as a marker of both undifferentiated and differentiating spermatogonia, while expression of DNMT3B identified differentiating spermatogonia^11,33^. The distinct stages of the seminiferous tubule areas were then defined according to the differentiation stage of resident spermatogonial populations as described^28,39^. Consistent with analysis of juvenile testis sections, the basal layer of adult *Sox3* null tubules demonstrated pronounced increases in the density of GFRα1-positive cells throughout the seminiferous epithelium cycle (Fig. 7A). Notably, while in WT tubules GFRα1-positive cells were most frequently present as A_s_ and A_pr_, in *Sox3* null testis they were often found as A_al_ chains of 4 or more cells. The increased GFRα1-positive chain length in *Sox3* null testis was accompanied by evidence of disruption to the spermatogonial differentiation pathway. Specifically, at stages II-V, when WT tubules contained abundant populations of GFRα1-/SALL4 + /DNMT3B- A_al_ cells; *Sox3*-deficient A_al_ often abnormally retained GFRα1 expression (Fig. 7A). Moreover, between stages V-XI, during which essentially all WT A_al_ cells initiate differentiation and generate GFRα1-/SALL4+/DNMT3B + differentiating A-type spermatogonia, a substantial fraction of A_al_ cells in the *Sox3*-deficient testis persisted in a GFRα1+/SALL4+/DNMT3B- undifferentiated state (Fig. 7A). Consequently, the density of differentiating spermatogonia at times appeared lower in *Sox3* null tubules as compared to WTs.

This data indicates that *Sox3*-deficient A_al_ cells, while morphologically resembling differentiation-primed undifferentiated spermatogonia do not appropriately down-regulate GFRα1, a key functional marker of the stem cell state^40^. Moreover, these GFRα1+ A_al_ cells subsequently enter the differentiation pathway less efficiently than their WT GFRα1– A_al_ counterparts. Consistent with our observations, the majority of GFRα1+ A_al_ that accumulated in *Sox3* null adults did not express RARγ (Fig. 7B), a marker of differentiation-primed progenitors required for differentiation commitment^17,18^. Interestingly, however, a minor subset of GFRα1+ A_al_ cells in the knockout did express RARγ, indicating an accumulation of transitional undifferentiated cells exhibiting both stem and progenitor characteristics. Such a stem-progenitor transitional state is rarely detected in WT adult testis, as is presumably short-lived but persists in the absence of *Sox3*, as undifferentiated cells appear unable to adopt a stable committed progenitor state (Fig. 7B). Note that RARγ + GFRα1– progenitors were still found in *Sox3* null testis indicating that stem to progenitor conversion was not completely abrogated, in agreement with our analysis of juvenile testis (Figs. 6B and 7B).

### *Ngn3* is a direct target of SOX3

**Figure 8:**
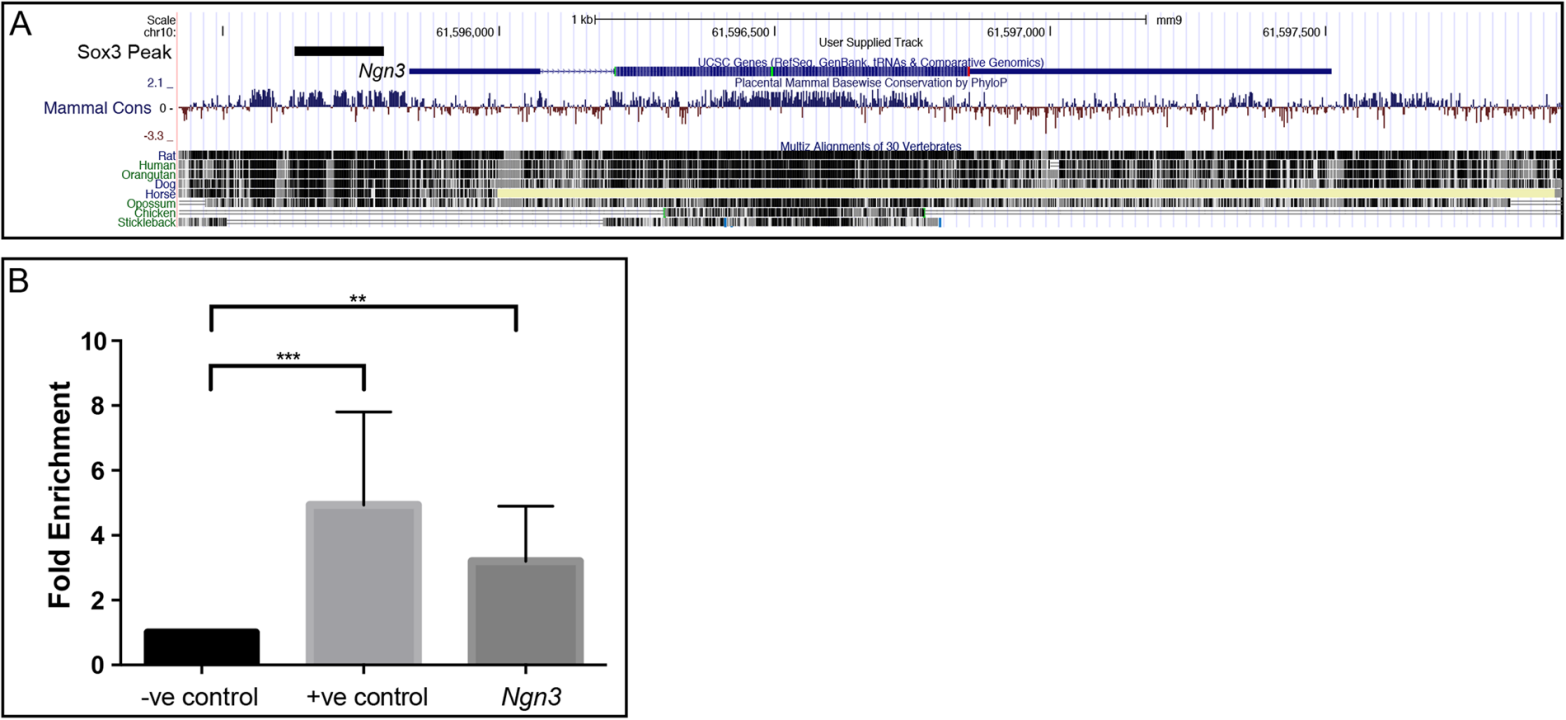
*Ngn3* is a direct SOX3 target gene in spermatogonia. UCSC Genome Browser view of published SOX3 ChIP-seq data^42^ from neural progenitor cells showing a SOX3 binding site within the upstream promoter region of *Ngn3* (**A**). ChIP-PCR for SOX3 in P7 testis (**B**), demonstrating SOX3 binding upstream of the *Ngn3* promoter (*Ngn3*), Ρ < 0.05 (*), Ρ < 0.01 (**) as determined by t-test, data represented as mean±s.d. –ve control represents a site within *Notch1* known not to bind SOX3, +ve control represents a site within intron 2 of *Dbx1* previously validated as a binding site of SOX3^41^.

Given the near complete failure to activate *Ngn3* expression in *Sox3* null postnatal testes, we investigated the possibility that *Ngn3* can be a relevant target of SOX3 in germline cells. Previous ChIP-Seq studies of neural progenitors^41,42^ identified a SOX3 binding peak and single SOX consensus motif (SOCM) approximately 500 bp upstream of the transcriptional start site of *Ngn3* (Fig. 8A). This genomic region is highly conserved in mammals suggesting it may function as an enhancer. To investigate SOX3 binding to this region, we performed SOX3 ChIP-PCR on P7 mouse testis. Significant enrichment of SOX3 binding to this region was detected, suggesting that *Ngn3* is directly activated by SOX3 *in vivo* (Fig. 8B). As a positive control, we confirmed that SOX3 in testis cells also binds the SOX3 target gene *Dbx1* previously identified in a neural system^43^.

## Discussion

Previous studies have shown that SOX3 is expressed in Type A spermatogonia^8,26,27^; however, these range from stem cells through to committed progenitors and overtly differentiating cells and it was unclear how the expression and function of SOX3 related to this progression. Here we show extensive overlap between SOX3 and multiple markers of undifferentiated spermatogonia (PLZF, LIN28A and SALL4) indicating that *Sox3* is expressed within this compartment. Comparison of SOX3 with spermatogonial stem cell markers GFRα1 and EOMES revealed limited or no overlap respectively, indicating that the SOX3-negative spermatogonial population are *bona fide* stem cells. Taken together, we conclude that *Sox3* is not expressed by the steady-state stem cell population (GFRα1-positive) but is rapidly upregulated during initial differentiation commitment steps of these cells. The lack of *Sox3* expression in DNMT3A/B, c-KIT and CCND1-positive A_3_ to B spermatogonia indicates that *Sox3* is subsequently downregulated at later stages of differentiation. Thus, *Sox3* expression closely mirrors that of *Ngn3*, a key marker of committed progenitor spermatogonia^44^. Definitive identification of *Ngn3-* expressing cells *in vivo* is hampered by the lack of a specific antibody and therefore requires knock-in GFP reporter mice. Therefore, given the availability of commercial specific antibodies, SOX3 provides a useful alternative marker for committed type A spermatogonia.

**Figure 9:**
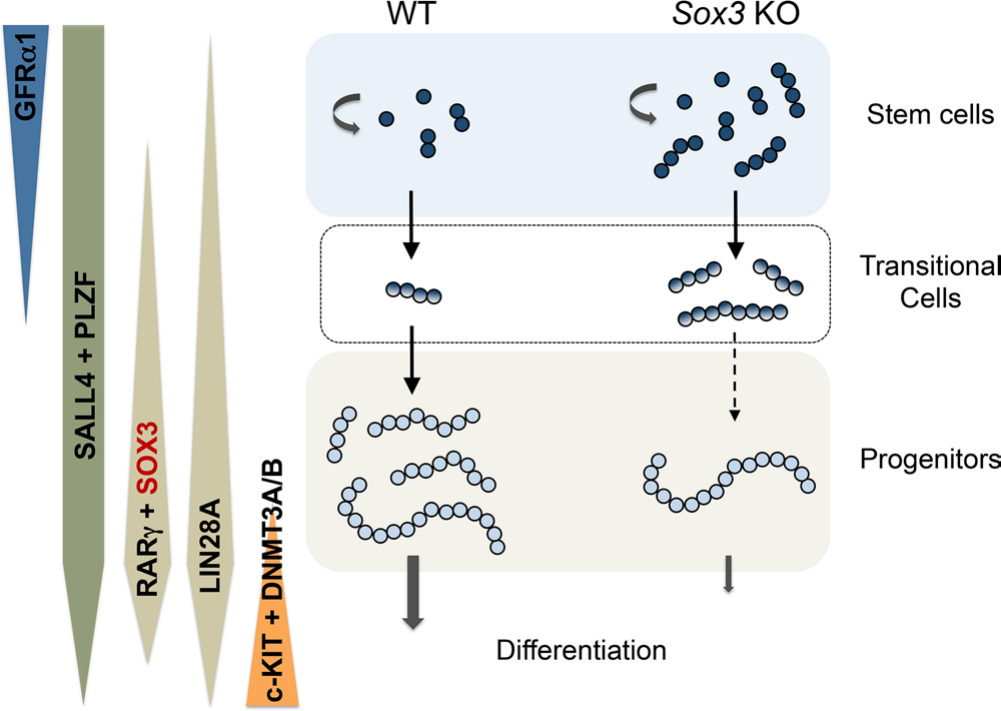
Model of spermatogenesis in WT and *Sox3* KO adult testis. GFRα1 is found within the spermatogonial stem cell pool of WT testis, before being switched off as the cells progress into a progenitor cell state. Conversely, SOX3 is not found within the stem cell population and expression is switched on upon conversion into progenitor cells. SOX3 + progenitor cells differentiate and continue through the spermatogenesis pathway. Additional markers delineating the distinct differentiation stages of spermatogonia are indicated. In *Sox3* knockout (KO) testis, the loss of SOX3 leads to an accumulation of GFRα1+ cells, and a reduction in the capacity to form differentiation-primed GFRα1- progenitor cells. This ultimately leads to a reduction in the production of differentiating spermatogonia. In adult WT testis, GFRα1+ stem cells are mostly present as A_s_ and A_pr_ while GFRα1+ A_al_ cells are frequently found in *Sox3* null testis. Stem-progenitor transitional A_al_ cells expressing both GFRα1 and RARγ also accumulate in *Sox3* knockouts but are rarely found in WT tubules. We propose that SOX3 plays an essential role in efficient stem to progenitor conversion within the undifferentiated spermatogonial population.

Within the undifferentiated spermatogonial pool, GFRα1+ cells have a high self-renewal potential while NGN3 + cells are primed to differentiate^14,21^. The distinct fates of these undifferentiated cell subsets are defined by relative sensitivity to retinoic acid (RA), a key endogenous regulator of spermatogonial differentiation. Specifically, NGN3 + cells are responsive to RA due to expression of the RA receptor RARγ, while GFRα1+ cells lack RARγ expression and are consequently unresponsive to RA^18,20^. Combined, our data demonstrate that SOX3 is critically required for formation of GFRα1-/NGN3 + differentiation-primed progenitors from the GFRα1 + /NGN3- stem cell pool. Moreover, we find that *Sox3* is specifically upregulated during this stem-to-progenitor transition and SOX3 directly targets *Ngn3* (see below). This suggests that SOX3 plays a central and cell-autonomous role in promoting exit from a GFRα1+ stem cell state. *Sox3* deletion therefore results in an accumulation of GFRα1+ cells and a substantially reduced capacity to generate RA-sensitive, differentiation-primed GFRα1- progenitors within the undifferentiated pool. Consequently, this leads to a reduction in production of differentiating spermatogonia and ineffective or blocked spermatogenesis (Fig. 9). In adults, loss of *Sox3* was also accompanied by an accumulation of transitional undifferentiated cells expressing both stem and progenitor markers, reflecting inefficient stem to progenitor conversion.

It is also possible that in the absence of SOX3, differentiation-primed GFRα1-negative progenitors are still successfully generated within the undifferentiated pool but are not stably maintained and rapidly revert to a GFRα1+ state, mimicking the cellular dynamics observed during testis regeneration^21^. This possibility could be tested by fate-mapping progenitor populations upon *Sox3* deletion, through use of *Ngn3-CreER* plus reporter lines or by developing an equivalent *Sox3-CreER* transgenic model^19^. Based on current models^13,21^, progenitor-to-stem cell reversion upon *Sox3* deletion would be predicted to be accompanied by fragmentation of longer undifferentiated progenitor chains back to A_s_ and A_pr_ GFRα1+ cells. However, in the *Sox3* null adult, we find GFRα1+ cell chain lengths to be increased compared to WT controls, arguing against this reversion model. Rather, our results suggest that the physiological process of differentiation priming that normally occurs as chain length increases within the undifferentiated pool does not occur in the *Sox3* null testis (Fig. 9). Consequently, a large proportion of the *Sox3*-deficient A_al_ population abnormally retains gene expression patterns and properties of the stem cell state, including being refractory to differentiation stimuli such as RA. This GFRα1+ A_al_ population generally lacks expression of RARγ, a marker of progenitor cells required for RA-dependent spermatogonial differentiation^17,18^.

We have provided evidence that *Ngn3* is a direct target gene of SOX3 in spermatogonia. NGN3 is a prototypical marker of the differentiation-destined progenitor state and knockdown of *Ngn3* expression in cultured undifferentiated spermatogonia disrupts differentiation upon transplantation *in vivo*^21,45^. Therefore, the loss of *Ngn3* expression upon *Sox3* deletion may be responsible for reduced efficiency of the stem cell population to progress to a committed progenitor state. Collectively our data define the window in which SOX3 acts during spermatogenesis, as well as one critical direct target gene, *Ngn3*. Further studies of both the regulation and the role of SOX3 will help reveal how type A spermatogonia progress from stem cells to committed precursors and the degree to which these processes are shared between spermatogonial and neuronal progenitors.

## Materials and Methods

### Animals

Generation of the *Sox3* null mice on a mixed genetic background (129 sv/eV, MF1, CBA and C57Bl/6) has been described previously^8^. Mixed background *Sox3* null mice were crossed onto the 129/SvJ genetic background and mice from the F6 generation or from a 129/SvJ, CBA mixed background were used in these studies.

### Testis histology and immunofluorescence (IF)

Testes were isolated and fixed in 4% formaldehyde overnight, followed by equilibration of tissues in 30% sucrose. Testis were set in OCT compound (Tissue-Tek) and cryosectioned on a Leica CM1900 at 16μm thickness. For IF, sections were permeablised with 1% Triton in PBS for 10 minutes, blocked with 10% horse serum for 1 hour, followed by overnight incubation with primary antibodies overnight at 4 °C. Sections were washed with PBS and incubated in secondary antibody solution for 1–2 hours, washed in PBS, mounted in ProLong Gold Anitfade Mountant with DAPI (Life Technologies, P-36931) before being cover slipped. Images were captured on a Nikon Ti-E inverted microscope with NIS software (NIKON) using a 20x objective lens. For histology, sections were dehydrated in an increasing methanol series from 10% to 100%. Sections were stained with haematoxylin and eosin. Whole mount IF of seminiferous tubules was performed as described^16,20^.

### Antibodies

Antibodies are detailed previously^16,20,30,31,33^_,_ including αSOX3 (R&D Systems AF2569), αDNMT3A (Novus Biologicals 64B1446), αDNMT3B (Novus Biologicals 52A1018), α c-KIT (Cell Signaling Technologies #3074), αLIN28A (Cell Signaling Technologies #8641) and α Cyclin D1 (Novus Biologicals SP4).

### TUNEL assay

TUNEL assays were performed as per manufacturer’s instructions (Roche, 11684795910). Briefly, tissue was permeabilised in 0.1% Triton X-100 for 2 minutes on ice, washed twice with PBS, then incubated with TUNEL reaction mixture for 60 mins at 37 C in a humidified chamber. Tissue was washed 3 times with PBS followed by mounting with ProLong Gold Anitfade Mountant with DAPI.

### qPCR

RNA from testis of all mouse ages was extracted by mechanically dissociating tissue in Trizol (Life Technologies) for 10 minutes as per manufacturer’s instructions. RNA was run on a 1.5% RNase free agarose gel to assess integrity. cDNA was generated using the High Capacity RNA to cDNA kit (Life Technologies). Expression profiling from total testis extracts was performed on four WT and four *Sox3* null testes at each age, using *B-Actin* and *Eif4a* as reference genes. RT-qPCR was performed using Fast Sybr Green Master Mix (Life Technologies), and run on the ABI 7500 StepOnePlus system, all reactions were completed in triplicate. Isolation and gene expression analysis of adult undifferentiated spermatogonia was performed as described^38^.

### Primer sequences

qPCR primers used for gene expression analyses were as follows:

*Sox3:* F 5′-GAACGCATCAGGTGAGAGAAG-3′, R 5′-GTCGGAGTGGTGCTCAGG-3′

*Gfra1:* F 5′-ATCGGGCAGTACACATCTCTG-3′, R 5′-TGTGGTTATGTGGCTGGAGG-3′

*Plzf:* F 5′-CCTGGACAGTTTGCGACTGA-3′, R 5′-GCCATGTCCGTGCCAGTAT-3′

*Ngn3:* F 5′-CCCCAGAGACACAACAACCT-3′, R 5′-AGTCACCCACTTCTGCTTCG-3′

*Oct4:* F 5′-CCCAGGCCGACGTGG-3′, R 5′-GATGGTGGTCTGGCTGAACAG-3′

*Id4:* F 5′*-*CTCACCCTGCTTTGCTGAGA-3′, R 5′-TCACCCTGCTTGTTCACGG-3′

*E-Cad:* F 5′-TTGCAAGTTCCTGCCATCCT-3′, R 5′-CATCATCTGGTGGCAGCAG-3′

*β****-****Actin:* F 5′-CTGCCTGACGGCCAGG-3′, R 5′-GATTCCATACCCAAGAAGGAAGG-3′

*Eif2*: F 5′-TGATGGCACTGGCCCCAACAT-3′, R 5′-GCGCCCTCCTTAGTAGCCCAC-3′

qPCR primers used for ChIP-PCR analyses were as follows:

*Ngn3:* F 5′-GAGAGTTGCTGGGACTGAGC-3′, R 5′-AGCTGGATTCCGGACAAAG-3′

*Dbx1:* (+ve control) F 5′-CTTTGGTCTCCACAAGCTTTCT-3′, R 5′-GAATGTGGCCTTTAACAACTCAC-3′

*Notch1:* (-ve control) F 5′-TGTTGTGCTCCTGAAGAACG-3′, R 5′-GCAACACTTTGGCAGTCTCA-3′

### ChIP-PCR

P7 mouse testis were extracted and tissue was mechanically disassociated in 1% formaldehyde for 8 minutes at room temperature, followed by neutralisation with 125 mM glycine for 5 minutes at 4 °C. Cells were lysed followed by sonication (Bioruptor, Diagenode) for 15 minutes with 30 s cycling pulses on ice. SOX3 bound chromatin was immunoprecipitated by a goat polyclonal antibody raised against human SOX3 (R&D systems, AF2569). DNA was recovered after reversing crosslinks and purified by PCR clean-up kit (QIAGEN). Three independent ChIP samples were generated each from the material of 4 testis. Each sample was analysed by qRT-PCR as described above, using 1% input as a reference and ChIP samples from *Sox3* null testis as a negative control.

### Ethics statement

Animal experiments were subject to approval by the Animal Ethics Committees of the University of Adelaide and Monash University. All studies were conducted within the principles of animal replacement and reduction and experimental refinement. Animals were monitored daily for evidence of illness and, if distressed, were culled immediately by cervical dislocation by an experienced investigator/animal technician. All the experiments were performed in accordance with the approved guidelines and regulations.

## Supplementary information

Supplementary Figures.
