# Supplementary material for: SOX3 promotes generation of committed spermatogonia in postnatal mouse testes
Source: Sci Rep. 2020 Apr 21;10:6751. doi: 10.1038/s41598-020-63290-3 (PMC7174399; doi:10.1038/s41598-020-63290-3)
Supplement: Supplementary file 1 — Supplementary Figures. [file 41598_2020_63290_MOESM1_ESM.docx]

**SOX3 promotes generation of committed spermatogonia in postnatal mouse testes.**

**Dale McAninch^1^, Juho-Antti Mäkelä^2,3,5^, Hue M. La^2,3,5^, James N. Hughes**^1^**, Robin Lovell-Badge^4^, Robin M. Hobbs^2,3,*^ and Paul Q. Thomas^1,*^**

^1^School of Biological Sciences and Robinson Research Institute, University of Adelaide, Adelaide, SA 5005, Australia

^2^Australian Regenerative Medicine Institute, Monash University, Melbourne, VIC 3800, Australia

^3^Development and Stem Cells Program, Monash Biomedicine Discovery Institute and Department of Anatomy and Developmental Biology, Monash University, Melbourne, VIC 3800, Australia

^4^Laboratory of Stem Cell Biology and Developmental Genetics, The Francis Crick Institute, London NW1 1AT

^5^These authors contributed equally

^*^Authors for correspondence ([paul.thomas@adelaide.edu.au](mailto:paul.thomas@adelaide.edu.au); robin.hobbs@monash.edu)

**Supplementary Figures**

**
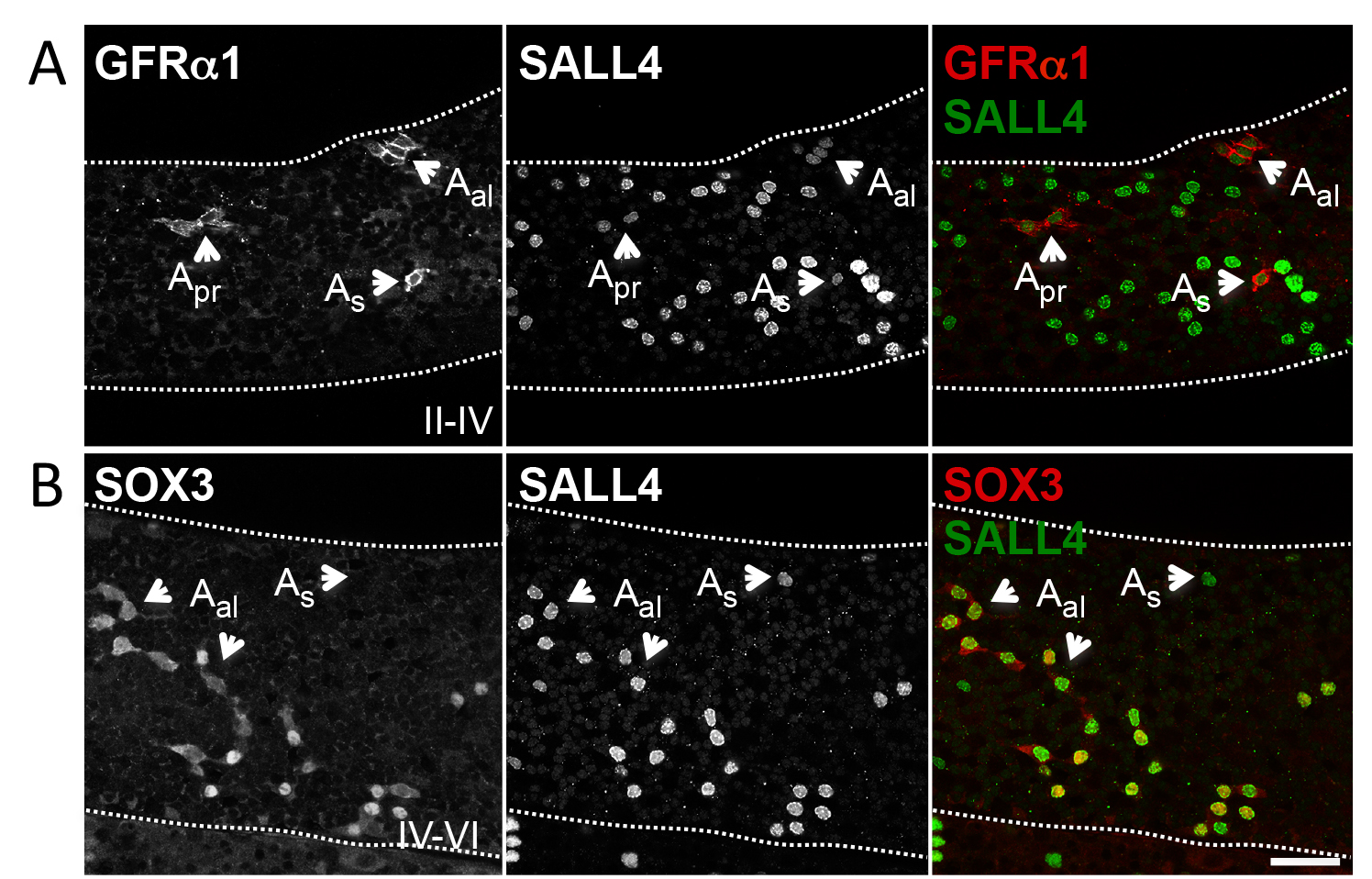
**

**Figure S1. Characterising expression pattern of SOX3 in adult testis.** Wholemount IF analysis of adult seminiferous tubules demonstrating distinct subcellular localisation patterns of GFRα1 (A) (cell membrane) and SOX3 (B) (predominantly nuclear) within undifferentiated spermatogonia. Demonstrates single stain antibody staining controls for GFRα1 + SOX3 co-stain within same fluorescence channel (antibodies are both raised in goat). Spermatogonia are counterstained with SALL4. Selected undifferentiated cells and stages of tubules are indicated. Representative images are shown (n = 4 mice). Scale bar, 50μm.


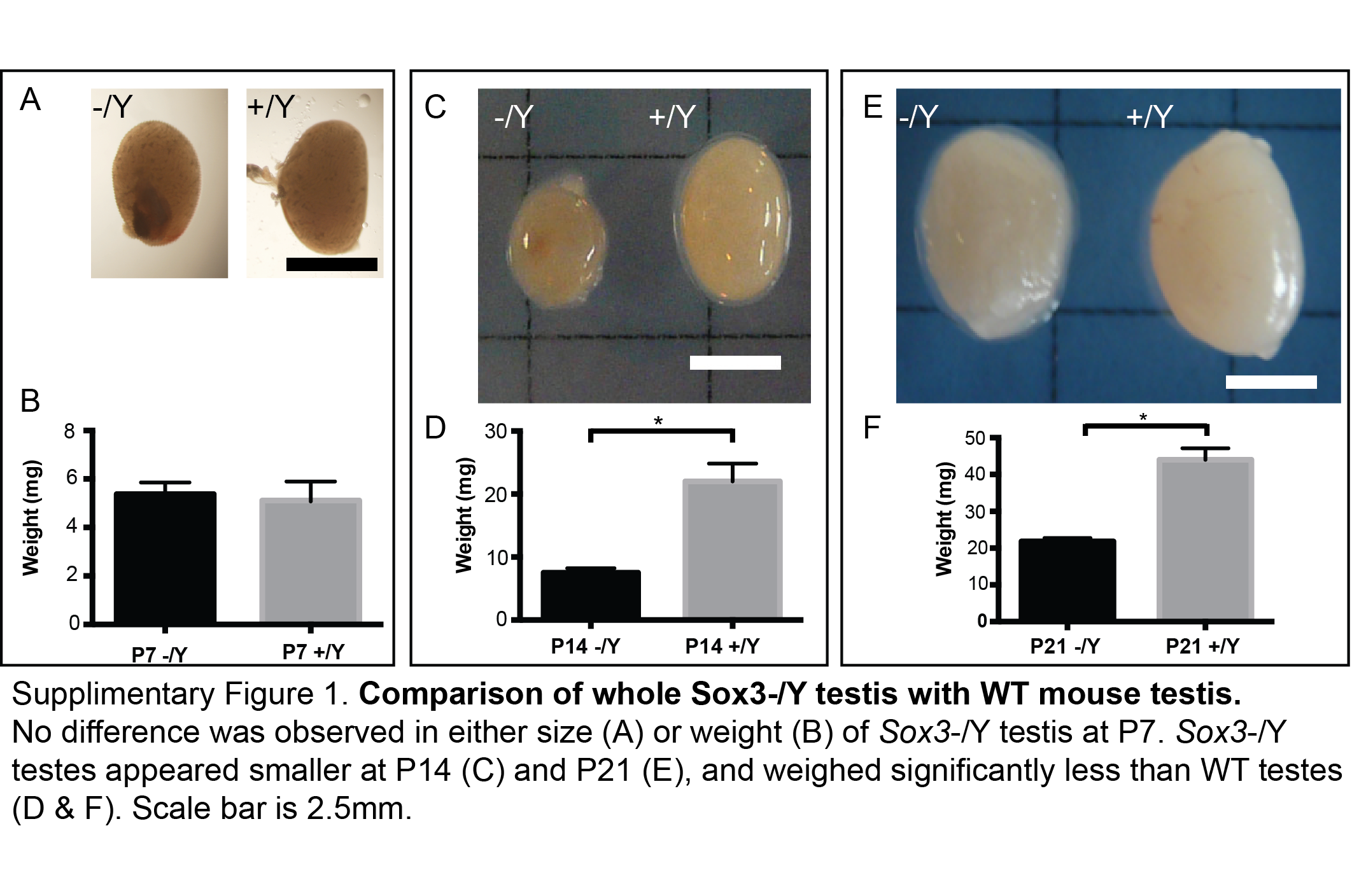


**Figure S2. Comparison of whole *Sox3* -/Y testes with WT mouse testes.** No difference was observed in either size (A) or weight (B) of *Sox3*-/Y testes at P7. *Sox3*-/Y testes appear smaller at both P14 (C) and P21 (E), and weigh significantly less than WT testes (D & F). Scale bars are 2.5mm.


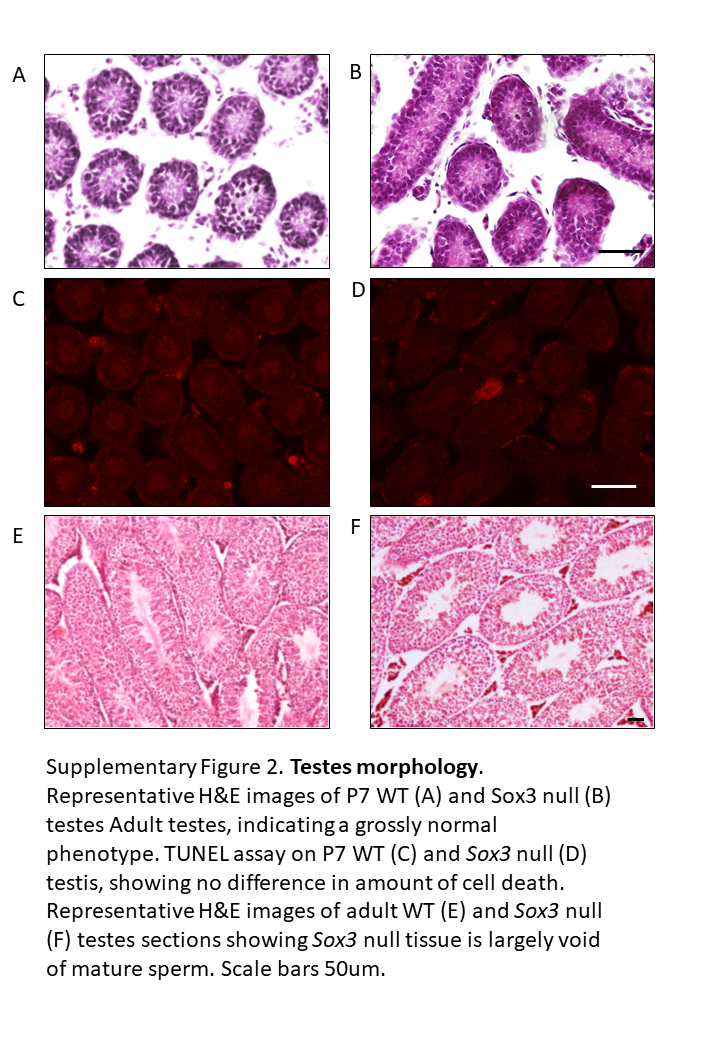


**Figure S3. Figure S3.** **Testes morphology**. Representative H&E images of P7 WT (A) and *Sox3* null (B) testes Adult testes, indicating a grossly normal phenotype. TUNEL assay on P7 WT (C) and *Sox3* null (D) testis, showing no difference in amount of cell death. Representative H&E images of adult WT (E) and *Sox3* null (F) testes sections showing *Sox3* null tissue is largely void of mature sperm. Scale bars 50um.
